# Intelligent large-scale flue-cured tobacco grading based on deep densely convolutional network

**DOI:** 10.1038/s41598-023-38334-z

**Published:** 2023-07-10

**Authors:** Xiaowei Xin, Huili Gong, Ruotong Hu, Xiangqian Ding, Shunpeng Pang, Yue Che

**Affiliations:** 1 Faculty of Information Science and Engineering, Ocean University of China, Qingdao, 266100 Shandong China; 2 School of Computer Engineering, Weifang University, Weifang, 261061 Shandong China; 3 Exhibition Department, Qingdao Revolutionary Martyrs Memorial Hall, Qingdao, 266071 Shandong China

**Keywords:** Plant sciences, Engineering

## Abstract

Flue-cured tobacco grading plays a crucial role in tobacco leaf purchase and the formulation of tobacco leaf groups. However, the traditional flue-cured tobacco grading mode is usually manual, which is time-consuming, laborious, and subjective. Hence, it is essential to research more efficient and intelligent flue-cured tobacco grading methods. Most existing methods suffer from the more classes less accuracy problem. Meanwhile, limited by different industry applications, the flue-cured tobacco datasets are hard to be obtained publicly. The existing methods employ relatively small and lower resolution tobacco data that are hard to apply in practice. Therefore, aiming at the insufficiency of feature extraction ability and the inadaptability to multiple flue-cured tobacco grades, we collected the largest and highest resolution dataset and proposed an efficient flue-cured tobacco grading method based on deep densely convolutional network (DenseNet). Diverging from other approaches, our method has a unique connectivity pattern of convolutional neural network that concatenates preceding tobacco feature data. This mode connects all previous layers to the subsequent layer directly for tobacco feature transmission. This idea can better extract depth tobacco image information features and transmit each layer’s data, thereby reducing the information loss and encouraging tobacco feature reuse. Then, we designed the whole data pre-processing process and experimented with traditional and deep learning algorithms to verify our dataset usability. The experimental results showed that DenseNet could be easily adapted by changing the output of the fully connected layers. With an accuracy of 0.997, significantly higher than the other intelligent tobacco grading methods, DenseNet came to the best model for solving our flue-cured tobacco grading problem.

## Introduction

As the primary raw material of the cigarette industry, flue-cured tobacco leaves affect the quality of tobacco formula products. Thus, improving the economic value of flue-cured tobacco is vital to meet cigarette industry development needs. The grading of flue-cured tobacco will result in the quality of the leaf, which will be bargained finally. Meanwhile, because different grades of flue-cured tobacco leaves have different chemical composition contents, and the amount of toxic substance (such as nicotine or CO), varies when different chemicals are burned, so flue-cured tobacco grading will affect the sensory evaluation quality and the smoke index that highly related to the smoker’s health^1^, while we should explore more explicit grading method to improve smoker's health. Therefore, flue-cured tobacco grading is significant to the national economy and smoker’s health^1^.

As we mentioned, it is a complicated task to realize intelligent flue-cured tobacco grading. Flue-cured tobacco leaves are divided into 42 grades in China according to seven factors: maturity, leaf structure, body, oil, color intensity, length, and waste. The traditional flue-cured tobacco grading mode mainly relies on human sensory methods (eyes and hands) to judge the appearance quality of tobacco leaves, which has two disadvantages: one is the traditional tobacco grading mode needs long-termly to train talents, which is time, cost, resources, and labor consuming. Another is the low classification accuracy caused by human subjectivity and instability.

All these problems seriously hinder tobacco leaf purchase and cigarette production. With the hot development of artificial intelligence (AI), AI technology has been applied in agriculture^2–5^, traffic engineering^6^ and industry^7,8^. For instance, in agriculture, feature enhancement and DMS-Robust Alexnet were used to identify maize leaf disease^9^, ABCK-BWTR and B-ARNet were combined to identify tomato leaf diseases^10^, and reference^11^ utilized CASM-AMFMNet to classify grape leaf diseases.

Hence, intelligent tobacco grading will be the trend in the future development of the tobacco industry. At present, the research on intelligent tobacco grading technology has achieved significant growth, mainly including three aspects:

1. The first way is using infrared^12^ and hyperspectral techniques, combined with stoichiometry to construct a classification model to achieve rapid and nondestructive tobacco classification^13–15^. However, the near-infrared equipment cost is high, and the spectrum it scans is more sensitive to environments (such as temperature and humidity), making classification results inaccurate.
2. Another way to realize tobacco grading is to establish decision rules based on fuzzy mathematics and chemical composition^16,17^. For example, in reference^16^, the classification accuracy was about 0.94 for the trained tobacco leaves, and the accuracy of the non-trained tobacco leaves was about 0.72. But this method has low accuracy because of the complex repeated reasoning.
3. The last way is computer vision solved by machine learning algorithms^18–22^, such as traditional machine learning methods (e.g. support vector machines (SVMs), random forests (RFs))^23^, deep learning methods^19,24^, and neural network^25^. Specially, due to the important economic status of tobacco, the industries have accumulated a large amount of tobacco data in the production process. But how to fully use these data and explore their value has become the trend of intelligent development in the tobacco industry. Deep learning can learn the rich internal rules and interpret information from the data^26^ and convolutional neural network (CNN) has strong feature extraction ability^27^. Therefore, deep learning is a crucial technology based on many labeled data for tobacco grading. Among them, the deep CNN has become the most critical technology to solve image recognition^28–31^. Due to the high effectiveness of CNN, such as Highway Networks^32^, Residual Networks (ResNets)^33^, Alexnet^28^, VGG^31^ etc., many scholars have done groundbreaking tobacco grading works through its variant structures^34,35^. For instance, in reference^19^, they fine-tuned a VGG16 network structure for tobacco grading. In reference^25^, a CNN classifier was used for tobacco grading leaves, and the accuracy was 0.9625. In reference^36^, a FDANet was proposed for flue-cured tobacco grading.

The methods mentioned above, although previously acknowledged, are still inadequate in terms of accuracy, rendering their practical application a challenge. Moreover, a predicament emerges wherein the accuracy dwindles as the number of classes increases. This phenomenon can be attributed to the insufficient feature extraction ability of deep convolutional neural networks caused by traditional connection mode of layers.

Hence, propelled by the large-scale flue-cured tobacco grade data accumulated by tobacco industries, we proposed an intelligent tobacco grading method based on DenseNet, surmounting the aforementioned quandaries. Namely, to extract tobacco grade characteristics deeply, we utilized DenseNet^37^ as a backbone. By concatenating preceding tobacco feature data, the connection mode of DenseNet is more conducive for extracting fine-grained specific visual information about tobacco leaves. The proposed method can make deep model thoroughly learned the sample distribution of each grade, thereby unlock the full potential of deep learning.

Moreover, based on the high-resolution professional data, we verified that the DenseNet structure performs better than other deep CNN structures on our high-quality tobacco grading datasets, improving the accuracy of tobacco grading.

In summary, our work is as follows:

1. We collected and sorted out large amounts of flue-cured tobacco grade data with high resolution and high usability that can support follow-up work. Meanwhile, we aim to reach enterprise-level publish for boosting industrial development.
2. We pre-processed the data using affine transformation, normalization, exponential moving average, etc., providing smoother data for better training. Then we designed the experiments for our dataset based on DenseNet.
3. We evaluated various classical methods on our flue-cured tobacco grade data, where the competitive experimental results demonstrated the high performance of DenseNet.

Our work can significantly reduce the use of the workforce and provide theoretical and technical support for the intelligent grading of tobacco leaves in the tobacco industry. Meanwhile, applying deep learning in tobacco can impact other industries such as agriculture and botany.

The remainder of this paper is structured as follows: “Methods” Section introduces the process of our method in detail. In “Experiments and results” Section, the experimental setup is given and the related results are discussed. Finally, “Conclusions” Section provides a summary of this paper as well as the scope of future work.

## Methods

**Figure 1:**
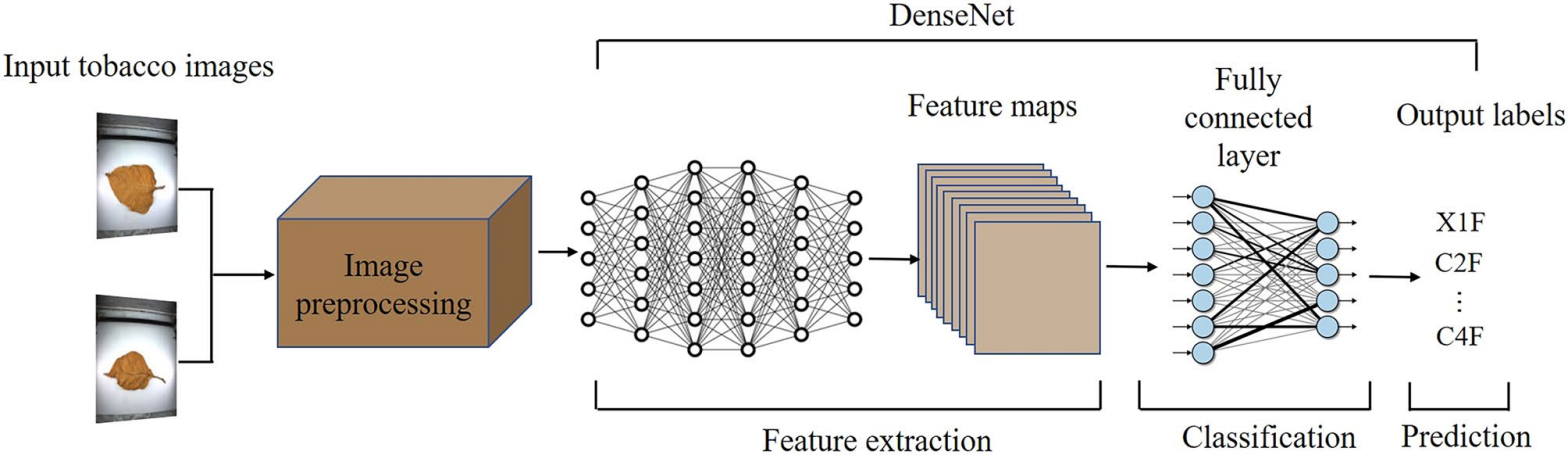
The framework of the tobacco grading process based on DenseNet.

In this section, we first introduce data acquisition, including experimental instruments and image acquisition method, and then propose the detailed pre-processing process for our dataset. Finally, we illustrate the idea of intelligent flue-cured tobacco grading based on DenseNet. Figure 1 shows the framework of our method.

As Fig. 1 shows, the tobacco grading process based on DenseNet includes training and test phases. In the training phase, the data was processed by pre-processing, feature extraction, classification, and prediction. While in the test phase, we first froze the parameters of the DenseNet model obtained in the training phase, then the test images were pre-processed and processed by the entire DenseNet model, including the feature extraction and classification. Finally, the model outputted the prediction results.

### Data acquisition

**Figure 2:**
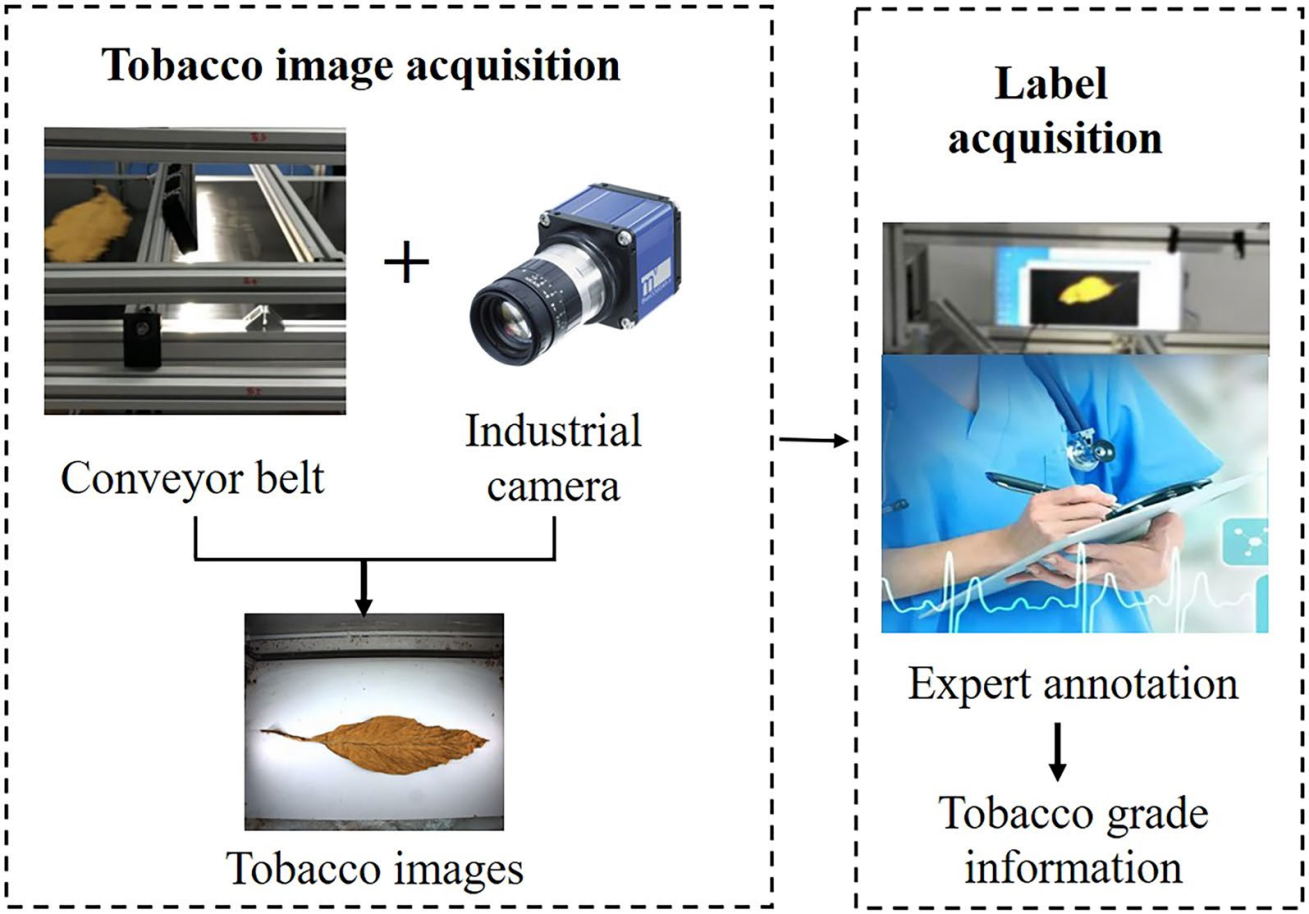
Pipeline of tobacco data acquisition.

To collect tobacco images, we chose the CMOS BV-C5400 line array industrial camera and the shot of BV-L1024 in our experiment. Table 1 shows the detailed configuration of our equipment and Fig. 2 illustrates the pipeline of tobacco data acquisition process.

**Table 1:** The parameter configuration of our equipment.

Equipment	Parameter types	Parameter settings
Industrial camera (BV-C5400)	Resolution Ratio	2456 × 2058
	Line Frequency	18.03 kHz
	Pixel Size	7 μm
	Sensor	CMOS
	Color	R, G, B
	Exposure Time	9.52 μs–100 ms
Shot (BV-L1024)	Sensor Length	30 mm
	Focal Length	f = 24 mm
	Aperture Range	F2.8–F22
	Focusing Range	0.4–2.0 m
	Applicable Pixel Size	7 μm

We can learn that data acquisition process includes image acquisition and label acquisition. Specifically, the tobacco samples were unfolded and laid flat on the conveyor belt first, and then the images were collected when the conveyor belt was running. Subsequently, industry experts marked the actual grading of tobacco samples according to the GB 2635–1992, a flue-cured tobacco grading standard in China.

**Figure 3:**
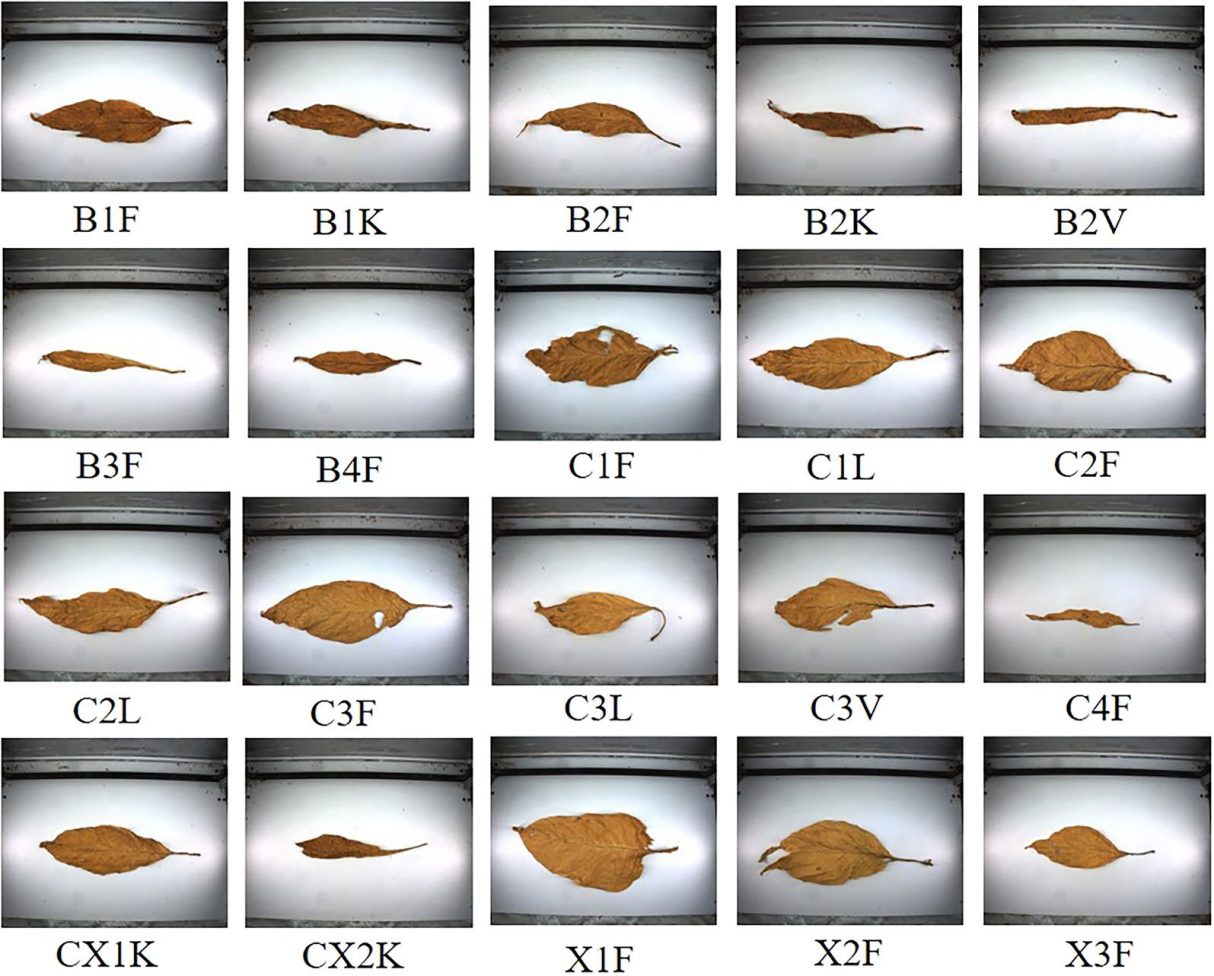
Data display of partial tobacco samples.

We chose 20 flue-cured grades that are the most widely used among 42 grades, including B1F, B1K, B2F, B2K, B2V, B3F, B4F, C1F, C1L, C2F, C2L, C3F, C3L, C3V, C4F, CX1K, CX2K, X1F, X2F, and X3F. Due to the significance of those grades, they can solve most flue-cured grading problems. We selected 21,113 representative tobacco images from the 20 grades to form our experimental dataset. Figure 3 shows some samples of the dataset.

Our dataset is a significant improvement both in quantity and quality compared to other datasets and it has been applied in the cigarette factory, produced specific effects, and improved grading efficiency.

### Data pre-processing

We divided the dataset into the train set and test set with the proportion of 8:2. Table 2 details the information on sample division.

**Table 2:** The information on tobacco samples division.

Grades*	Training set	Test set	Total number
B1F	1102	276	1378
B1K	770	192	962
B2F	1016	254	1270
B2K	699	175	874
B2V	677	169	846
B3F	974	244	1218
B4F	763	191	954
C1F	943	236	1179
C1L	621	155	776
C2F	1102	275	1377
C2L	589	147	736
C3F	1030	258	1288
C3L	1070	267	1337
C3V	653	163	816
C4F	950	237	1187
CX1K	746	187	933
CX2K	535	134	669
X1F	664	166	830
X2F	1091	272	1363
X3F	896	224	1120

∗ indicates: According to China’s current flue-cured tobacco grading standard, we divide flue-cured tobacco leaves into 42 grades. The grades of our dataset are the subset of the 42 grades.

**Figure 4:**
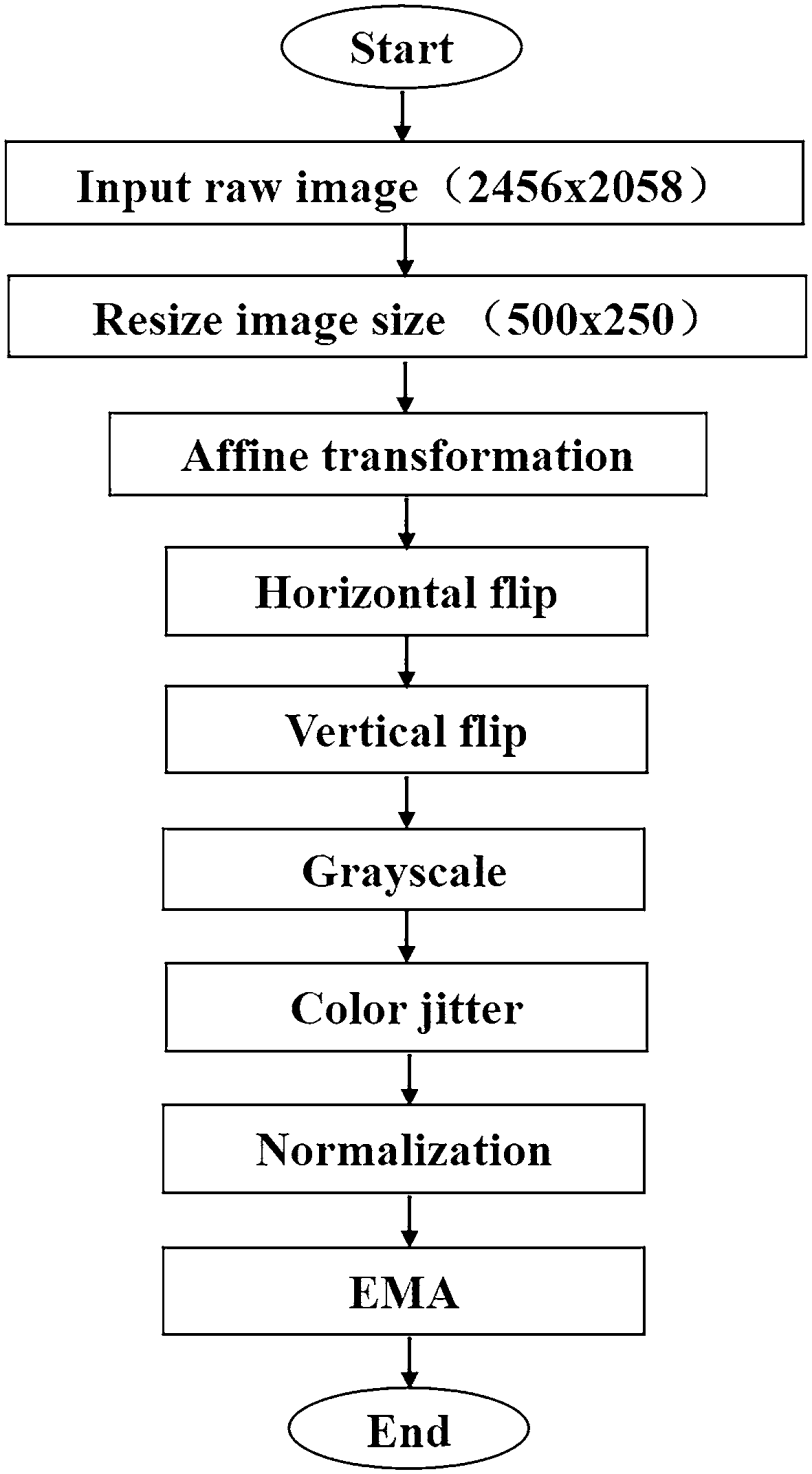
The process of pre-processing data includes resize, affine transformation, horizontal flip, vertical flip, grayscale, color jitter, normalization, and EMA.

**Figure 5:**
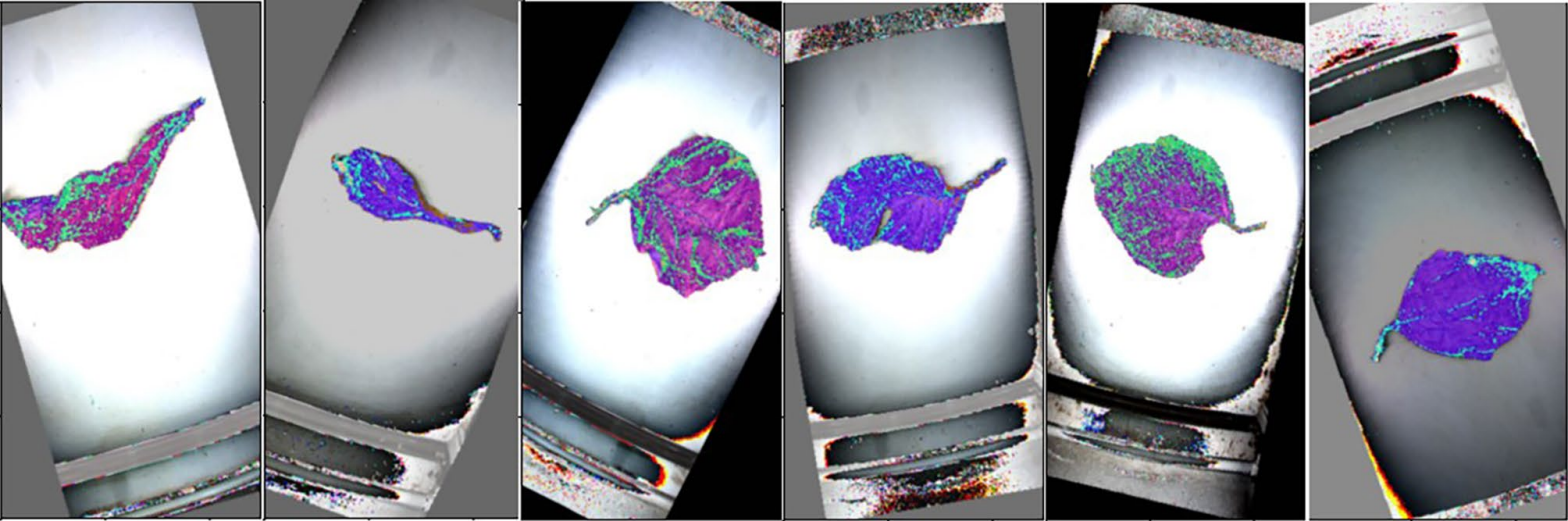
Visualization of some tobacco leaf images after preprocessing.

To make the original data more suitable for neural network processing and fully extract features, the original data must undergo pre-processing operations, including rotation, translation, normalization, and others before model training. Figure 4 shows the image pre-processing process, and Fig. 5 visualizes some tobacco leaf images after preprocessing.

In our experiment, the input size of the original image was 2456 × 2058, and the image was scaled to 500 × 250 as actual input for better training. Subsequently, we operated the image by an affine transformation, horizontal random rotation, vertical flip, and transformation to a grayscale image. We used the Cross-Entropy (CE) as the loss function, which mainly describes the distance between the actual and expected output probabilities. In other words, the smaller the Cross-Entropy value is, the closer the two probability distributions are. Assuming that the probability distribution $p\left( x \right)$ is the expected output and the probability distribution $q\left( x \right)$ is the actual output, then the solution of Cross-Entropy Loss is shown in Eq. (1):

1 $$ \begin{array}{*{20}c} {L\left( {p,q} \right) = - \mathop \sum \limits_{x} p\left( x \right){\text{log}}q\left( x \right)} \\ \end{array} $$

The normalization operation is required to facilitate subsequent data processing and ensure faster convergence during program run time.

Exponential moving average (EMA), also called weighted moving average, is an averaging method that gives more weight to recent data. The significance of EMA lies in using moving average parameters to improve the model on the test data of robustness. The moving average can be considered the mean value of the variables in the past period. Compared with direct assignment for a variable, getting the value of the moving average on the image is smoother, less jittered, and reduces fluctuations.

For instance, for the variables of $\left[ {\theta_{1} ,\theta_{2} ,\theta_{3} , \ldots ,\theta_{n} } \right]$, where $\theta_{1} ,\theta_{2,} \theta_{3,} \ldots \theta_{n}$ represent the pixel values of tobacco images. The ordinary way to get the average is as follows:

2 $$ \begin{array}{*{20}c} {avr = \frac{1}{n}\mathop \sum \limits_{i = 1}^{n} \theta_{i} } \\ \end{array} $$

In Eq. (2),$$ avr$$ represents the average of n variables. However, $$avr_{k} $$ in EMA is computed as follows:

3 $$ \begin{array}{*{20}c} {avr_{k} = \alpha \cdot avr_{k - 1} + \left( {1 - \alpha } \right) \cdot \theta_{k} } \\ \end{array} $$

In Eq. (3),$$ avr_{k}$$ represents the average of the first k pieces of data, $$\alpha$$ is the weight value (generally set as 0.9–0.999), which is set as 0.999 in this paper. Consequently, the $$avr_{k}$$ is the input for the neural network.

### Deep convolutional neural network

#### Convolutional neural network

**Figure 6:**
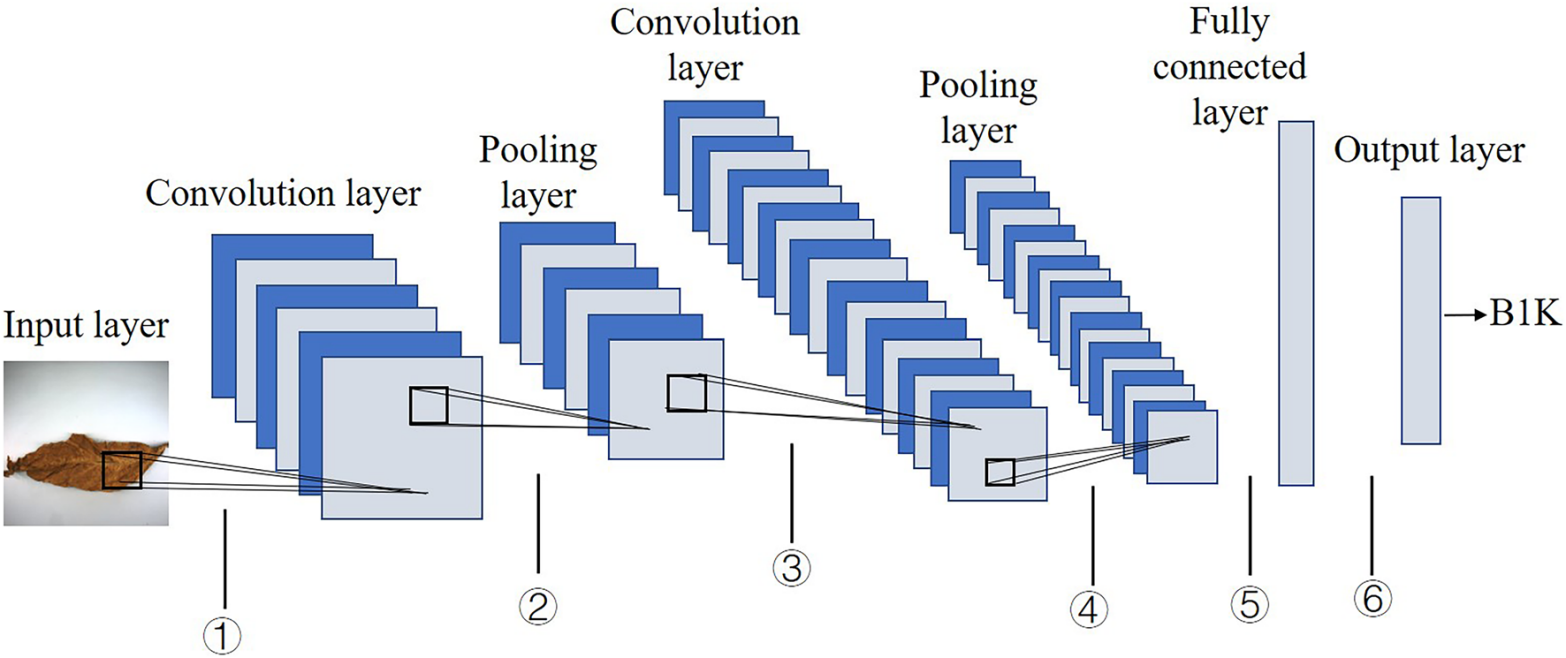
The structure of convolutional neural network. ①, ③ indicate convolution operation, ②, ④indicate pooling, ⑤ indicates fully connecting, ⑥ indicates softmax process.

DenseNet is one of the convolutional neural networks, and the structure of the convolution process is illustrated in Fig. 6. Processing by multi-convolution and pooling layers, the images are transformed into helpful feature maps, then fully a connected layer combined with the Softmax layer results in the grade prediction.

#### Flue-cured tobacco feature extraction based on DenseNet

Traditional connection mode of a deep convolutional neural network summarizes preceding feature data when the data pass into a network layer. This mode can lead to vanishing-gradient and poor reuse of feature data problems. These problems result in the insufficiency of feature extraction ability for different grades of tobacco leaves. In contrast to other methods, DenseNet combines data by concatenating them. So traditional convolutional networks with ${\text{L}}$ layers have ${\text{L}}$ connections—one between each layer and its subsequent layer—while DenseNet has $\frac{{{\text{L}}\left( {{\text{L}} + 1} \right){ }}}{2}$ direct connections. The detailed process of proof is shown in reference^37^. As a result, flue-cured tobacco feature extraction based on DenseNet alleviated the vanishing-gradient problem, encouraged feature reuse, and substantially strengthened the feature extraction ability. Therefore, we utilized DenseNet as a backbone to extract tobacco leaf features and automatically classify tobacco leaves.

The details of the DenseNet connection are as follows: assume that $X_{0}$ is the input single tobacco leaf image, the output of layer L is $X_{l}$, and the neural network has $L$ layers totally, and each layer will go through a transition layer such as Batch Normalization (BN), rectified linear units (ReLU), Pooling, or Convolution (Conv), then we define the nonlinear transformation operation layer is $F_{l} \left( \cdot \right)$. In $F_{l} \left( \cdot \right)$, $l$ denotes the index number of layers.

In a traditional deep convolutional neural network, take the output of layer $l{\text{th}}$ as the input of layer $\left( {l + 1} \right){\text{th}}$. The layer transition calculation method of $l{\text{th}}$ layer and $\left( {l - 1} \right){\text{th}}$ layer is shown in Eq. (4), so the $l{\text{th}}$ layer only receives the output from one previous layer.

4 $$ \begin{array}{*{20}c} {X_{l} = F_{l} \left( {X_{l - 1} } \right)} \\ \end{array} $$

**Figure 7:**
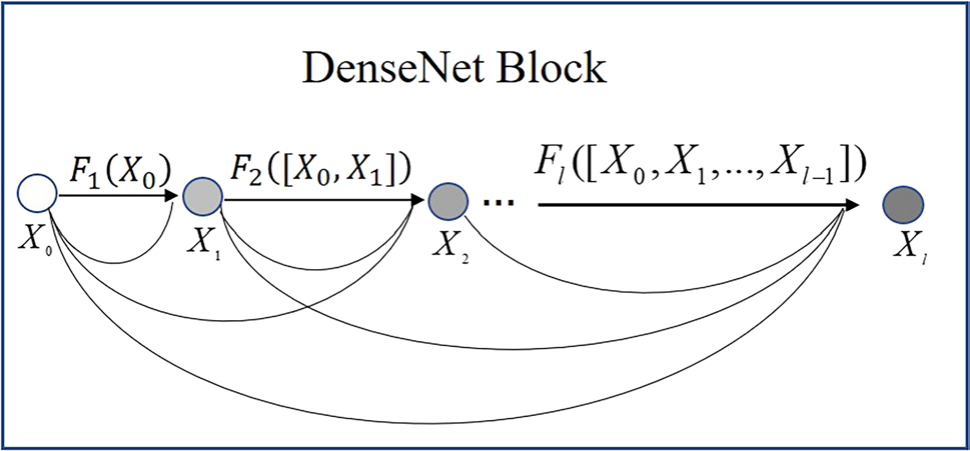
The connection pattern of layers and the information flow of DenseNet. The $l{\text{th}}$ layer receives the feature maps of all preceding layers.

This information flow mode may impede the data transmission in the network. However, DenseNet has a different connectivity pattern. They connect any layer to all subsequent layers directly. Figure 7 illustrates the connection pattern of layers and information flow.

As Fig. 7 shows, the $l{\text{th}}$ layer receives the feature maps of all preceding layers. This connection mode can better carry out feature extraction and better transmit each layer's features, reducing the loss of feature information. Specifically, the feature transmission mode of DenseNet is shown in Eq. (5).

5 $$ \begin{array}{*{20}c} {X_{l} = F_{l} \left( {\left[ {X_{0} ,X_{1} , \ldots ,X_{l - 1} } \right]} \right)} \\ \end{array} $$

In Eq. (5), $\left[ {X_{0} ,X_{1} ,...,X_{l - 1} } \right]$ refers to the concatenation of the feature-maps produced in layers 0 to $l - 1$, (i.e. $1 + 2 + 3 + \cdots {\text{L}} = \frac{{L\left( {L + 1} \right)}}{2}$), so they introduce $\frac{{L\left( {L + 1} \right)}}{2}$ connections to the network. Compared with other deep convolutional neural networks, the feature transmission mode of DenseNet can better extract depth image information features.

## Experiments and results

In this section, we first illustrate our experiment environment and experimental hyperparameter settings and then introduce our experiments and the results.

### Experiments

#### Experiment settings

Our experiment was based on python 3.8, PyTorch 1.8 environment, and a GPU of Tesla V100. First, we carried out flue-cured tobacco grading experiments on the traditional machine learning methods (SVM, RF, KNN, LightGBM and XGBoost). Table 3 shows the grid search hyperparameters and the optimal parameters for 20 grades of traditional machine learning methods.

**Table 3:** Grid search hyperparameters and optimal parameters of traditional methods.

Methods	Hyperparameters	Search space	Optimum parameters
SVM	C	1, 10, 100, 1000	1
	Gamma	0.0001, 0.001	0.001
	Kernel	linear, rbf	linear
RF	Criterion	gini, entropy	entropy
	Max_depth	4, 5, 6, 7, 8	8
	Max_features	auto, sqrt, log2	auto
	n_estimators	200, 300, 500	500
KNN	n_neighbors	2, 3, 4, 5,…,19	17
	Weights	Uniform, distance	distance
LightGBM	Learning_rate	0.001, 0.01, 0.1	0.1
	Max_depth	4, 6, 8	8
	Min_child_samples	2, 3, 5	5
	Num_leaves	6, 9, 12	9
	Reg_alpha	0, 0.01, 0.03	0
XGBoost	Eta	0.001, 0.01, 0.1	0.01
	Gamma	0.0001, 0.001, 0.01, 0.1	0.001
	Max_depth	3, 6, 9,12	9

Then, we used DenseNet and other deep models to verify the validity of the dataset. Table 4 is the hyperparameter settings for deep models. In deep learning, hyperparameters are parameters that need to be set manually before the model is trained. They are not automatically learned from the training data, but are set by humans. The selection and fine-tuning of these hyperparameters have an important effect on both the performance and training process of the model. Specifically, hyperparameters can control the complexity of model. For instance, in Table 5, different numbers represent different network structures of DenseNet. Moreover, Hyperparameters can affect the training process and convergence rate of the model. For example, the learning rate determines the stride size of each model parameter update. Too high or too low a learning rate may lead to instability or convergence difficulties in the training process. Besides, the setting of hyperparameters can help prevent the model from overfitting. For example, in Table 4, a normalization parameter is a common hyperparameter that plays an important role in data pre-processing, improving feature comparison and weight balance, accelerating model convergence, preventing numerical overflows, and enhancing model generalization.

**Table 4:** Hyperparameter settings of deep models.

Numbers	Hyperparameters	Settings
1	Resize	500 × 250
2	ColorJitter_brightness	0.4
3	ColorJitter_contrast	0.5
4	ColorJitter_saturation	0.5
5	ColorJitter_hue	0
6	Normalize_mean	0.5
7	Normalize_std	0.5
8	EMA	0.999
9	Weight_decay	0.00025
10	Initial Learning Rate	1e−3
11	Batch Size	16
12	Output Dropout	0.1
13	Epochs	150

**Table 5:** The test accuracy of different DenseNet structures.

DenseNet Models^37^	Accuracy (10epoch)	Accuracy (20epoch)	Accuracy (50epoch)	Accuracy (70epoch)	Accuracy (80epoch)	Accuracy (100epoch)	Accuracy (150epoch)
DenseNet121	0.882	0.946	0.980	0.986	0.989	0.992	0.995
DenseNet161	0.961	**0.984**	**0.995**	**0.996**	**0.996**	0.996	0.996
DenseNet169	0.943	0.975	0.992	0.993	0.993	0.994	0.995
DenseNet201	**0.963**	0.983	0.994	0.994	0.995	**0.996**	**0.997**

Significant values are in [bold].

Nevertheless, the selection and adjustment of hyperparameters is an iterative and time-consuming process. It requires comprehensive consideration and adjustment in relation to specific problems, data sets and models. At the same time, the optimal value of the hyperparameter is not fixed and may depend on the task.

In Table 4, Numbers from 1 to 9 are the parameter settings of image pre-processing. The main purpose of the operation is to eliminate irrelevant information, recover useful real information, enhance the detectability of relevant information and simplify the data to the maximum extent, so as to better fit the deep model structures and improve the reliability of feature extraction and recognition. Numbers 10–13 are the parameter settings of model training. It is worth noting that the settings of the value are all through repeated experiments, experience and observations. And they are most suitable for our experimental environment and the results obtained by using these parameters are optimal.

#### Evaluation metrics

The used evaluation metrics are computed by Eqs. (6–9). Here, TP is “True Positive”, it means true is 0 and prediction is 0; FN is “False Negative”, it means true is 0 and prediction is 1, FP is “False Positive”, it means true is 1 and prediction is 0, TN is “True Negative”, it means true is 1 and prediction is 1.

① Accuracy

Equation (6) shows the overall Accuracy of the classification model (for all classes), here $N$ is the number of all samples.

6 $$ \begin{array}{*{20}c} {{\text{Accuracy }} = \frac{TP + TN}{N}} \\ \end{array} $$

② Precision

Precision reflects the ability of the model to distinguish negative samples. The higher the Precision, the stronger the ability of the model. Precision is computed by Eq. (7).

7 $$ \begin{array}{*{20}c} {{\text{Precision }} = \frac{TP}{{TP + FP}}} \\ \end{array} $$

③ Recall

On the contrary, Recall reflects the ability of the model to recognize positive samples. The higher the Recall, the stronger the ability of the model. Recall is calculated by Eq. (8).

8 $$ \begin{array}{*{20}c} {{\text{Recall }} = \frac{TP}{{TP + FN}}} \\ \end{array} $$

④ F1-Score

Combining the metrics of Precision and Recall, the value range of F1-Score is from 0 to 1, where 1 is the best and 0 is the worst. The higher the F1-score, the more robust the model. The computing method of F1-Score is shown as Eq. (9).

9 $$ \begin{array}{*{20}c} {F1 - {\text{Score }} = \frac{{2*P{\text{recision }}*{\text{ Recall }}}}{{{\text{ Precision }} + {\text{ Recall }}}}} \\ \end{array} $$

### Results and analysis

**Figure 8:**
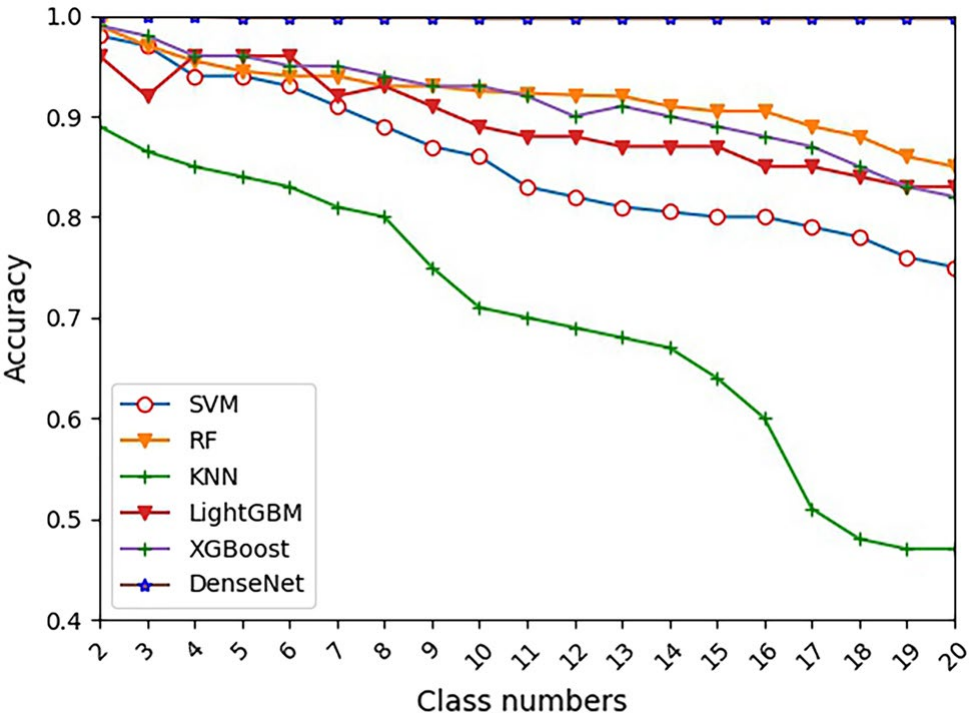
The accuracy of different classes for different methods on the test dataset.

We first used accuracy as the evaluation indicator of different methods. Figure 8 is the accuracy of different number of tobacco grades for traditional methods. It suggests that an increase of tobacco grade categories significantly decreases the classification accuracy of traditional machine learning methods (SVM, RF, KNN, LightGBM and XGBoost). Therefore, when the number of tobacco grades is large, traditional machine learning methods are not suitable for tobacco grading. However, the accuracy of DenseNet is significantly higher and more stable than others. Moreover, Table 5 shows the performance of flue-cured tobacco grading based on different DenseNet structures on the test dataset.

**Figure 9:**
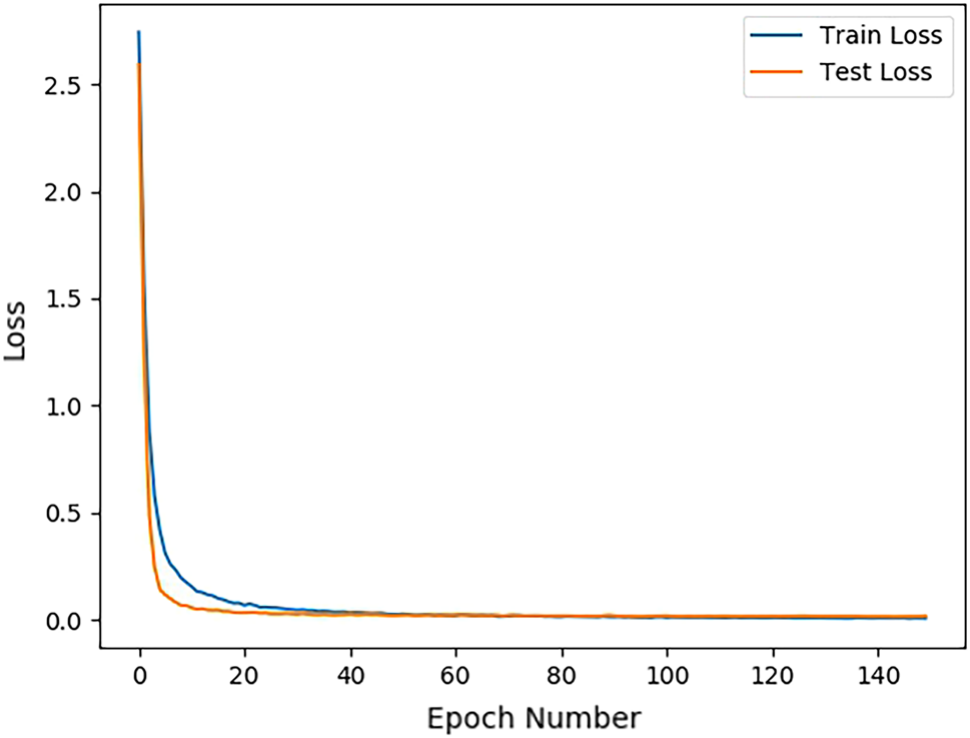
The loss of DenseNet169.

As shown in Table 5, the flue-cured tobacco grading results of different DenseNet structures are slightly different, but all are above 0.995, and Fig. 9 is the loss of DenseNet169 during the training process.

To verify the grading accuracy of DenseNet, we compared DenseNet with other methods, including traditional machine learning and deep learning methods, and the results are Tables 6 and 7. Here, “Accuracy without preprocessing” represents the experimental results without preprocessing. From Tables 6 and 7, we can learn that our preprocessing on the final result is positive, it can improve the accuracy to some extent. Besides, the final accuracy of tobacco classification based on DenseNet has reached 0.997, which is significantly higher than others. DenseNet can find more subtle and general features among tobacco grades and dig more individual features for tobacco, so flue-cured tobacco grading based on DenseNet performs better than others.

**Table 6:** The accuracy of DenseNet versus traditional machine learning methods.

Methods	Accuracy without preprocessing	Accuracy
KNN^38^	0.40	0.47
RF^39^	0.81	0.85
SVM^3^	0.72	0.75
LightGBM^40^	0.79	0.83
XGBoost^41^	0.78	0.82
DenseNet^37^	**0.951**	**0.997**

Significant values are in [bold].

**Table 7:** The accuracy of DenseNet versus deep learning methods.

Deep models	Epoch	Accuracy without preprocessing	Accuracy
ResNet-18^33^	600	0.683	0.729
ResNet-34^33^	600	0.910	0.953
Resnext50_32 × 4d^33^	400	0.901	0.939
ResNet-101^33^	300	0.943	0.984
Resnext101_32 × 8d^33^	300	0.932	0.975
ResNet-152^33^	300	0.968	0.985
VGG16^31^	200	0.674	0.710
VGG19^31^	260	0.872	0.939
MobileNet^42^	600	0.840	0.936
Fine-tuned VGG16^19^	300	0.868	0.947
DarkNet19 & K-Means^24^	400	0.812	0.854
DRSN^12^	300	0.918	0.934
FDANet^36^	500	0.807	0.856
DenseNet^37^	150	**0.951**	**0.997**

Significant values are in [bold].

From Tables 6 and 7, we can see deep learning methods perform better than traditional machine learning methods. Besides, as the number of network layers increases, the convergence is faster and the grading performance becomes better.

In order to prove the advantages of our proposed method from different views, we utilized more evaluation indicators, including precision, recall and F1-score. Table 8 illustrates the results. We can learn that tobacco grading based on DenseNet still significantly outperforms the other methods.

**Table 8:** The precision, recall and F1-score of DenseNet versus other methods.

Models	Precision	Recall	F1-score
KNN^38^	0.476	0.451	0.439
RF^39^	0.853	0.846	0.861
SVM^3^	0.762	0.756	0.758
LightGBM^40^	0.844	0.827	0.829
XGBoost^41^	0.832	0.871	0.826
ResNet-152^33^	0.970	0.963	0.982
VGG19^31^	0.931	0.923	0.935
MobileNet^42^	0.940	0.916	0.932
Fine-tuned VGG16^19^	0.945	0.912	0.941
DarkNet19 & K-Means^24^	0.856	0.840	0.861
DRSN^12^	0.927	0.914	0.938
FDANet^36^	0.859	0.871	0.846
DenseNet^37^	**0.981**	**0.989**	**0.996**

Significant values are in [bold].

Hence, we can conclude that DenseNet has almost entirely learned the image features of different grades of flue-cured tobacco leaves, so it can accurately identify each flue-cured tobacco grade.

### Discussion

Although our proposed method outperforms other tobacco grading approaches, it has its limitations. Firstly, our method requires a large amount of labeled data, but obtaining enough supervised data is laborious and time-consuming and sometimes it is almost impossible due to privacy, safety or ethic issues. Therefore, the scarcity of data poses a significant challenge for constructing an excellent model. Furthermore, deep neural networks have requirements in terms of hardware and computation time, which leads the model is not as easy to implement. Nonetheless, we remain confident that with the right resources and expertise, our method will make contributions to the tobacco grading.

## Conclusions

This paper used DenseNet to fully extract flue-cured tobacco image information and achieved intelligent tobacco grading with high accuracy. Compared with traditional intelligent tobacco leaf classification technologies, such as SVM, RF, KNN, and other deep learning models, the efficiency and stability have significantly improved.

Our research has provided a promising solution for large-scale flue-cured tobacco grading and yielded remarkable results that have theoretical and practical implications. From a theoretical standpoint, our research enriches the existing theoretical system of tobacco grading and provides new insights into the fundamental methods and models. Besides, our work has a deeper understanding of the relationship between deep neural networks and their connection mode with model performance, which provides an idea for subsequent research in theory. For example, when the accuracy of tobacco leaf grading is not ideal, more samples or changing connection mode of the network can be attempted. On the other hand, the practical implications of our research are significant. For instance, our method can be used by tobacco enterprise to develop new grading tools that can improve the efficiency and effectiveness of their work. Meanwhile, it can also promote the standardization and unified development of tobacco leaf purchase to a certain extent, which can save costs and improve the economic benefits of enterprises ultimately. Besides, it can also provide new ideas for other fields, such as the agriculture, planting industry and medical domain, and promote the landing of artificial intelligence technology.

In future work, we plan to investigate other advanced tobacco grading methods and study more lightweight networks to reduce the computational time when training models, ensuring the balance between the accuracy and computational cost. On the other hand, we will promote the practical applications of our method for tobacco enterprise such as online intelligent grading system to improve efficiency. Furthermore, we will try to apply our proposed algorithm to more applications such as medical images identification and agricultural disease detection.

## Data Availability

The datasets generated and/or analysed during the current study are not publicly available because the raw data is currently private, but it is available from the corresponding author on reasonable request.
